# Supplementary material for: Time-series transcriptome analysis identified differentially expressed genes in broiler chicken infected with mixed Eimeria species
Source: Front Genet. 2022 Aug 8;13:886781. doi: 10.3389/fgene.2022.886781 (PMC9393255; doi:10.3389/fgene.2022.886781)
Supplement: Supplementary file 2 [file DataSheet1.ZIP › 7dpi_GO.Gsea.1625106495923/GOBP_REGULATION_OF_TRANSLATION_NCRNA_MEDIATED.html]

Details for gene set GOBP\_REGULATION\_OF\_TRANSLATION\_NCRNA\_MEDIATED[GSEA]

|  || Dataset | TMM\_7dpi\_gct\_format\_7dpi\_gct\_format.Class\_4dpi.cls #PC\_versus\_NC.Class\_4dpi.cls #PC\_versus\_NC\_repos |
| Phenotype | Class\_4dpi.cls#PC\_versus\_NC\_repos |
| Upregulated in class | 0 |
| GeneSet | GOBP\_REGULATION\_OF\_TRANSLATION\_NCRNA\_MEDIATED |
| Enrichment Score (ES) | -0.7501325 |
| Normalized Enrichment Score (NES) | -1.8829118 |
| Nominal p-value | 2.6329648E-4 |
| FDR q-value | 0.07705102 |
| FWER p-Value | 0.5268 |
Table: GSEA Results Summary

  

Fig 1: Enrichment plot: GOBP\_REGULATION\_OF\_TRANSLATION\_NCRNA\_MEDIATED      
 Profile of the Running ES Score & Positions of GeneSet Members on the Rank Ordered List

  

| SYMBOL | TITLE | RANK IN GENE LIST | RANK METRIC SCORE | RUNNING ES | CORE ENRICHMENT || 1 | EIF6 | na | 2557 | 0.184 | -0.1907 | No |
| 2 | EIF4G1 | na | 4628 | -0.064 | -0.3554 | No |
| 3 | EIF4E2 | na | 5204 | -0.122 | -0.3885 | No |
| 4 | AGO1 | na | 6589 | -0.264 | -0.4716 | No |
| 5 | DDX6 | na | 9932 | -0.698 | -0.6647 | Yes |
| 6 | TNRC6A | na | 9943 | -0.702 | -0.5797 | Yes |
| 7 | TRIM71 | na | 10125 | -0.736 | -0.5048 | Yes |
| 8 | TNRC6C | na | 10138 | -0.739 | -0.4154 | Yes |
| 9 | EIF4ENIF1 | na | 10321 | -0.774 | -0.3359 | Yes |
| 10 | AGO4 | na | 10509 | -0.812 | -0.2521 | Yes |
| 11 | STAT3 | na | 11052 | -0.950 | -0.1811 | Yes |
| 12 | TNRC6B | na | 11311 | -1.042 | -0.0752 | Yes |
| 13 | AGO3 | na | 11427 | -1.090 | 0.0486 | Yes |
Table: GSEA details [plain text format]

  

Fig 2: GOBP\_REGULATION\_OF\_TRANSLATION\_NCRNA\_MEDIATED      
 Blue-Pink O' Gram in the Space of the Analyzed GeneSet

  

Fig 3: GOBP\_REGULATION\_OF\_TRANSLATION\_NCRNA\_MEDIATED: Random ES distribution      
 Gene set null distribution of ES for **GOBP\_REGULATION\_OF\_TRANSLATION\_NCRNA\_MEDIATED**
